# Mitochondrial Neurogastrointestinal Encephalomyopathy: Novel Pathogenic Mutation in Thymidine Phosphorylase Gene in a Patient from Cape Verde Islands

**DOI:** 10.1155/2019/5976410

**Published:** 2019-12-11

**Authors:** Catarina Falcão de Campos, Miguel Oliveira Santos, Rafael Roque, Isabel Conceição, Mamede de Carvalho

**Affiliations:** ^1^Department of Neurology, Department of Neurosciences and Mental Health, Hospital de Santa Maria, Centro Hospitalar Universitário Lisboa Norte, Lisbon, Portugal; ^2^Instituto de Medicina Molecular and Instituto de Fisiologia, Faculdade de Medicina, Universidade de Lisboa, Lisbon, Portugal; ^3^Neuropathology Unit, Department of Neurosciences and Mental Health, Hospital de Santa Maria, Centro Hospitalar Universitário Lisboa Norte, Lisbon, Portugal

## Abstract

Mitochondrial Neurogastrointestinal Encephalomyopathy (MNGIE) is a rare autosomal recessive disorder caused by mutations in the gene encoding the Thymidine Phosphorylase (TP). It is clinically characterized by severe gastrointestinal dysmotility, cachexia, palpebral ptosis, ophthalmoparesis, sensorimotor polyneuropathy and leukoencephalopathy. The diagnosis is established by the presence of typical clinical and neuroimaging features, positive family history, and abnormal genetic test. A 19-year-old Cape Verdean patient with a history since childhood of recurrent episodes of nausea, vomiting, diarrhoea and painful abdominal distension associated with progressive motor disability with difficulty in climbing stairs and running and clumsiness with her hands. The diagnostic workup was suggestive of MNGIE. Genetic screening of the *TYMP* gene identified a novel mutation (c. 1283 G>A). Patients with MNGIE have significant comorbidity and mortality, and they are frequently misdiagnosed. A better acknowledgment of this disorder is essential to permit an earlier diagnosis and to improve disease management.

## 1. Introduction

Mitochondrial disorders (MDs) are a complex group of neuromuscular diseases in which the main features derive from mitochondrial dysfunction. Their prevalence has been difficult to establish and it is not yet known, mainly due to its clinical and genetic heterogeneity. Recent studies suggest that MDs are more frequent than previously considered, with an estimated prevalence of 1/4300 [1]. MDs heterogeneity can be explained by the fact that mitochondria metabolism requires two genomes: mitochondrial DNA (mtDNA) and nuclear DNA (nDNA), with a maternal and Mendelian inheritance, respectively [2].

Mitochondrial Neurogastrointestinal Encephalomyopathy (MNGIE) is a rare autosomal recessive disorder, and is considered as a classic example of MD secondary to a defect in the intergenomic communication [3]. Its prevalence is unknown; fewer than 100 cases have been reported. MNGIE is caused by loss of function mutations in *TYMP* gene, a nuclear gene encoding thymidine phosphorylase (TP), located on chromosome 22 [4]. TP initiates the catabolism of the pyrimidine nucleosides thymidine (dThd) and deoxyuridine (dUrd) by catalyzing the phosphorolysis of both nucleosides to deoxyribose phosphate and the corresponding bases, namely thymine and uracil [5]. In MNGIE patients, TP activity is very low resulting in systemic accumulation of dThd and dUrd, mitochondrial nucleotide pool imbalances with secondary depletion, multiple deletions and point mutations of mtDNA [6]. There is also evidence that TP activity is critical for angiogenic activity, which may further contribute to the pathophysiological mechanism of MNGIE [5].

MNGIE is clinically characterized by ptosis, external ophthalmoplegia, severe gastrointestinal dysmotility, cachexia, sensorimotor polyneuropathy and leukoencephalopathy. This disorder typically has an onset before the age of 30 years, associated with a severe prognosis and high mortality rate between the ages of 20 and 40 years [7]. Recently, allogeneic haematopoietic stem cell transplantation (HSCT) and orthotopic liver transplantation (OLT) have been proposed as a disease-modifying treatment [8, 9].

We report a young woman from Cape Verde origin, with a clinical diagnosis of MNGIE and a novel homozygous mutation in association with a frequent synonymous polymorphism in the *TYMP* gene.

## 2. Case Report

A 19-year-old female born and living in Cape Verde islands was admitted with a history of recurrent episodes of nausea and vomiting, gastric regurgitation, diarrhoea and painful abdominal distension, since 5 years of age. Those episodes became more frequent and severe in late adolescence resulting in a significant weight loss (≈15 kg) over the previous year. In association, she complained of slowly progressive gait impairment, difficulty in climbing stairs and inability to run. She referred poor gymnastic classes performance in school. She also mentioned progressive weakness of upper limbs with functional impact in combing her hair, writing and using cutlery. There was no history of cognitive impairment and academic achievement was good. She rejected pain, sensory or other dysautonomic symptoms. She was the second daughter of a consanguineous marriage. Her older sister had a similar clinical picture with gastrointestinal complaints; she received the diagnosis of superior mesenteric artery syndrome.

She developed cachexia (Body Mass Index of 10.9). On neurological examination, she had mild bilateral symmetric ptosis with no ocular paresis, generalized muscle weakness and atrophy, more pronounced in lower extremities and in distal segments, absent tendon reflexes, decreased nociception below knees, reduced vibration and position sense in toes and fingers, and required bilateral support to walk.

Routine laboratory studies were unremarkable, including creatine kinase and thyroid hormone levels. Plasma lactate level was above normal range (2.83 mM/L, normal 0.5–1). CSF analysis disclosed 2.5-fold increased protein content with normal number of cells; lactate level was also increased (2.7 mmol/L, normal 0.88–1.4).

Upper gastrointestinal endoscopy showed gastric aperistalsis associated with major dilation and ulcerative esophagitis. A nasojejunal feeding tube was required for enteral feeding.

Nerve conduction studies revealed marked demyelinating sensorimotor polyneuropathy (Table 1) and needle electromyography showed a superimposed myopathic pattern in proximal muscles.

**Figure 1:**
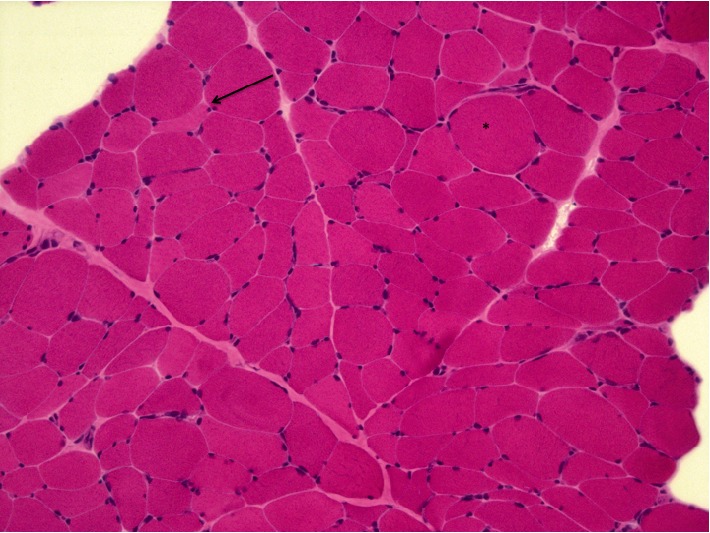
Muscle biopsy H&E × 10. It is observed increased variation in fiber size, with round hypertrophic fibres (^∗^) associated with angular atrophic fibres (arrow). No mitochondrial changes were detected.

Patient's muscle biopsy of the deltoid muscle disclosed unspecific myopathic changes such as type I fibre predominance and increased variation in fiber size with small, angular muscle fibres and round hypertrophic fibers. No signs of mitochondrial dysfunction (ragged red fibers, Cox-negative fibers and increased oxidative staining on SDH) were seen (Figure 1).

**Figure 2:**
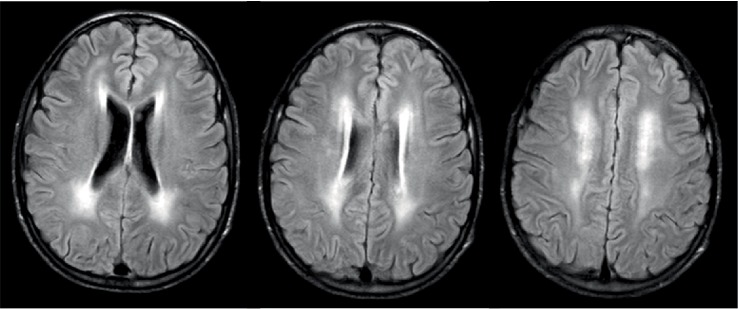
Brain MRI. Axial FLAIR images show symmetric and confluent increase signal intensity in deep white matter.

Brain MRI revealed unspecific symmetric and confluent T2-hyperintensity images in the deep white matter (Figure 2).

The presence of severe gastrointestinal dysmotility associated with a demyelinating sensorimotor polyneuropathy and myopathic changes suggested a mitochondrial disorder, specifically MNGIE. Genetic screening of the TYMP gene identified two homozygous contiguous mutations (c. 1283G>A, c.1284 T>A), affecting the same codon (GGT>GAA), causing together cause the amino acid change p.Gly428Asp. Prediction tools of pathogenicity (Polyphen, SIFT, Provean, SNP&GO) agreed with the damaging role of this genetic change. Unfortunately, DNA samples from family members were not available to perform segregation study.

The patient died three months after admission from medical complications associated with poor absorption and profound cachexia.

## 3. Discussion

We report a 19-year-old Cape Verdean woman with typical clinical features of MNGIE syndrome in whom we identified a novel homozygous *TYMP* gene mutation.

The gastrointestinal symptoms were the most prominent manifestation resulting in gastric atony, poor absorption and cachexia. Her older sister, living in Cape Verde islands, had milder but similar gastrointestinal symptoms. She was diagnosed as “superior mesenteric artery syndrome”, which is a common misdiagnosis in patients with MNGIE in the early course of the disease[7]. Their parents were asymptomatic but consanguineous, indicating an autosomal recessive transmission.

Patient poor functional ability was probably related to the presence of a marked demyelinating polyneuropathy. Indeed some cases of MNGIE syndrome have been misdiagnosed as chronic demyelinating inflammatory neuropathy [10]. It is hypothesized that the pathogenic mtDNA accumulates in the peripheral nerve, causing segmental and focal defects [10]. However, proximal weakness and ptosis were probably associated with the muscle fibres dysfunction observed in mitochondrial myopathies.

The main laboratory findings in these patients are plasma lactic acidosis and high CSF protein, as seen in our patient [7]. Muscle biopsy typically shows morphological and biochemical findings suggestive of mitochondrial abnormalities, such as the presence of ragged red fibers and Cox-deficient fibers. In our case, the muscle biopsy was not helpful. To our knowledge, there are few described MNGIE cases with normal muscle biopsy [7]. Therefore, a normal muscle biopsy does not exclude a MNGIE syndrome, in particular in young subjects. As reported elsewhere, our patient had symmetrical confluent T2-hyperintensity in the deep white matter. However, these changes do not impair cognition [11].

The genetic screening confirmed the diagnosis. Two homozygous contiguous mutations (c. 1283G>A, c.1284 T>A), affecting the same codon (GGT>GAA) were identified. To our knowledge, the first mutation has never been described in literature. The second is a frequent synonymous polymorphism. However, both mutations together caused the amino acid change p.Gly428Glu [12]. Although we were not able to perform TP activity and dThd and dUrd serum levels measurement and family segregation study in order to confirm its pathogenicity, bioinformatics genetic analysis (Polyphen, SIFT, Provean, SNP&GO) suggested a pathogenic role for this genetic change. Also, the associated typical patient's phenotype, compatible with MNGIE syndrome, further supported its pathogenicity.

Different therapeutic strategies have been proposed for patients with MNGIE syndrome. Some attempts were made to reduce the toxic accumulation of the nucleoside levels (dThd and dUrd) with dialysis and platelets transfusion, since platelets are rich with TP. However, both these approaches are not feasible as long-term therapies because there is an accumulation of the nucleosides [13, 14]. Halter and his colleagues showed that allogeneic HSCT could be an effective disease-modifying treatment. However, it was associated with a non-negligible mortality [8]. Additionally, OLT has been proposed as a new treatment in patients with MNGIE since the liver may be a tissue source of TP. OLT was successfully performed in two patients with early normalization of nucleosides levels with no serious adverse events [9]. Whether this finding is associated with a long-term quality of life improvement and increased survival remains to be elucidated. However, both these treatments may be considered in carefully selected patients, before severe organ damage occurs, making crucial an early diagnosis of MNGIE in these patients. Unfortunately, our patient was admitted in a late stage of the disease, dying few months later. More recently, treatment with adeno-associated virus vector containing the *TYMP* coding sequencing targeting the liver showed a persistent reversion of biochemical abnormalities in murine models without adverse effects. This observation indicates that in the future gene therapy may also be a feasible and efficient therapeutic strategy for MNGIE patients [15].

To date, more than 50 mutations have been reported. Here, we report a novel mutation (c. 1283G>A) in a patient with severe gastrointestinal dysmotility and polyneuropathy phenotype.

## Figures and Tables

**Table 1:** Patient's nerve conduction studies (NCS).

Motor NCS	Latency (ms)	Amplitude (mV)	Velocity (m/s)	F-Wave
*Median*		5.4	**28.4** (NV>50)	Absent
Wrist	4.9			
Elbow	12.3			
*Ulnar*		4	**24.4** (NV>50)	Absent
Wrist	4.1			
Elbow	12.7			
*Peroneal*		0.4	**15.4** (NV>40)	Absent
Ankle	4.8			
Fibula (below)	11.3			

Sensory NCS	Latency(ms)	Amplitude(*μ*V)	Velocity(m/s)	

Median	Absent			
Ulnar	Absent			
Radial	Absent			
Sural	Absent			
